# Lupus-Associated Pulmonary Arterial Hypertension: Variable Course and Importance of Prompt Recognition

**DOI:** 10.1155/2015/328435

**Published:** 2015-07-02

**Authors:** Kofi A. Mensah, Rajwardhan Yadav, Terence K. Trow, Cristina M. Brunet, Wassim H. Fares

**Affiliations:** ^1^Section of Rheumatology, Department of Internal Medicine, Yale University School of Medicine, 333 Cedar Street, New Haven, CT 06510, USA; ^2^Section of Pulmonary, Critical Care and Sleep Medicine, Department of Internal Medicine, Yale University School of Medicine, 333 Cedar Street, New Haven, CT 06510, USA

## Abstract

We describe a critically ill young woman with systemic lupus erythematosus (SLE) presenting with circulatory shock, multiorgan dysfunction, and elevated right-sided heart pressures. She was found to have recurrent acute severe pulmonary arterial hypertension (PAH) in the setting of an SLE flare. Our report highlights the variable course that SLE-associated PAH can take in the same patient and the implications of this for instituting the most effective treatment approach with each episode. This report also highlights the potential for SLE-associated PAH to present with life-threatening symptoms requiring critical care level interventions. We also describe evidence-based therapies, which can result in significant improvement in symptoms, function, and long-term outcomes.

## 1. Introduction

Pulmonary arterial hypertension (PAH) is a potentially lethal manifestation of systemic lupus erythematosus (SLE). Pulmonary hypertension (PH) is defined as an elevated mean pulmonary arterial pressure (mPAP) of 25 mmHg or greater at rest, and it is a heterogeneous condition with multiple underlying etiologies. The World Health Organization (WHO) has classified PH into five categories (Table 1) [1]. In total, the prevalence in the general population of these five classes of PH is unknown but has been estimated recently to be up to 10–20%, depending on the method of diagnosis and the population studied, with WHO group 2 (related to left heart failure) being the most common [1, 2]. The clinical severity of PH can be graded according to the WHO functional class system (Table 1).

## 2. Case Report

A 25-year-old woman with SLE presented in the spring of 2014 with hypotension and hypoxemia. She was in her usual state of health until two weeks prior to presentation, when she noticed worsening generalized body aches, malaise, excessive daytime sleepiness, and progressive shortness of breath with exertion. She described open sores in her mouth, productive cough, palpitations, chest pain, abdominal pain, lower extremity swelling, and diffuse joint pain. She denied fevers or chills. She took prednisone 60 mg daily at the onset of her symptoms.

Her past medical history was significant for SLE diagnosed in 2011, when she developed arthralgia, rash, and Raynaud's phenomenon. She had a positive anti-nuclear antibody (ANA) and proteinuria, and renal biopsy showed WHO V glomerulonephritis. She was initially treated with cyclophosphamide and then maintained on 200 mg hydroxychloroquine twice daily and 60 mg oral prednisone daily. She had been hospitalized in 2012 with an SLE flare and severe WHO group 1 SLE-associated PAH, which presented in a manner similar to the 2014 presentation described in this case report. She was treated with pulse methylprednisolone and intravenous cyclophosphamide in 2012 without specific pulmonary vasodilator therapy, gradually recovered, and was discharged home on prednisone 60 mg daily and hydroxychloroquine 200 mg twice daily. She was nonadherent to her medications and clinic visits.

On physical exam at the 2014 admission, she was afebrile, hypoxemic with an oxygen saturation of 89% on room air, and hypotensive with a BP of 73/52 mmHg. She had shallow, nonbleeding, ulcerated hard palate lesions. Pulmonary auscultation revealed decreased breath sounds and tachypnea. She had distended neck veins, regular tachycardia (135 beats/minute), and a prominent second pulmonic heart sound. Her abdomen was tender to palpation. There was no synovitis or joint effusion. Her fingers and toes were cold and clammy with a purplish hue and skin mottling. She had no focal neurological deficits.

**Figure 1:**
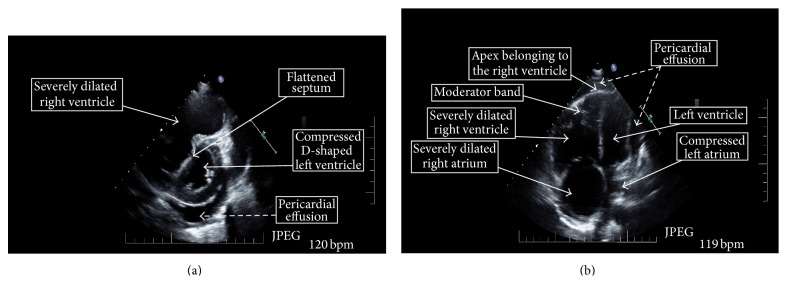
Echocardiogram of the patient demonstrating key features of severe pulmonary arterial hypertension. (a) Left parasternal short axis view. (b) Four-chamber apical view. Elevated pulmonary artery systolic pressures lead to a dilated right ventricle and right atrium. Dilation of the right ventricle causes flattening of the interventricular septum and the normally larger left ventricle becomes constricted. The rapid heart rate of 120 bpm also reduces the time for left ventricular filling and coronary artery perfusion. All of this results in hemodynamic compromise with decreased cardiac output, which can result in cardiogenic shock.

A computerized tomographic angiogram of the chest was negative for pulmonary embolus and lung parenchymal disease. A V/Q scan was low probability for pulmonary embolus without moderate or large segmental mismatched perfusion defects. ECG showed a new right bundle branch block. Chest X-ray revealed cardiomegaly. Transthoracic echocardiogram (TTE) showed a moderate to large pericardial effusion without tamponade, severe right atrial enlargement and tricuspid regurgitation, a plethoric inferior vena cava with decreased respiratory variation consistent with increased right atrial pressure, severe right ventricular (RV) enlargement, with severe RV systolic dysfunction, and RV wall hypokinesis. The RV systolic pressure (RVSP) was estimated at 70 mmHg. The left ventricle cavity size was decreased with interventricular septal flattening in systole and diastole consistent with RV pressure and volume overload (Figure 1).

The patient was admitted to the medical intensive care unit for management. Laboratory analysis revealed evidence of tissue hypoperfusion with severe anion-gap metabolic acidosis (pH 7.17, anion gap of 21 mmol/L, and serum bicarbonate of 8 mmol/L), central venous oxygen saturation of 49%, and multiorgan failure manifested by a creatinine 83.9 *μ*mol/L (up from 38.1 *μ*mol/L at baseline), AST/ALT 3350/1150 U/L (normal range 0–34), and INR 2.2 (in the absence of anticoagulant therapy). Her inflammatory and autoimmune markers confirmed an SLE flare with hypocomplementemia and elevation in anti-dsDNA titer (Table 2). Extensive infectious work-up, including blood cultures, was negative. Given the constellation of these clinical findings (and the absence of an infection, acute loss of circulating blood volume, or central nervous system insult), she was diagnosed with acute cardiogenic shock and started on vasopressors and inotropes.

More than a week into her hospitalization, her SLE flare was improving but definitely not resolved as she continued to be hemodynamically unstable requiring vasopressors and inotropes (though at lower dosages). Thus, right heart catheterization (RHC) was performed at that time (while still on inotropes and vasopressors) to guide further PAH- and RV-directed therapies. The RHC showed markedly worse hemodynamic parameters compared to values from 2012, when a RHC was done after she received immunosuppressive therapy for a similar presentation (Table 3). Given these clinical and hemodynamic findings she was diagnosed as WHO group 1 PAH, which is associated with connective tissue diseases such as systemic sclerosis and SLE. The severity of her clinical presentation placed her in WHO functional class IV, which is characterized by symptoms with any physical activity or while at rest (Table 1).

## 3. Discussion

The prevalence of PAH in patients with SLE is unclear with several studies suggesting a range from 0.5 to 43%, though 0.5 to 17.5% is reported in more recent studies [1, 3, 4]. The variation in reported prevalence may be related to the methods used to diagnose PAH, specifically the use of echocardiography versus the gold standard of RHC [4]. Echocardiography provides a noninvasive screening method, but the accuracy of echocardiography is about 50% in estimating RVSP [5]. As a screening method, echocardiography has a sensitivity of 50–90% and a specificity of 75–96% when compared to RHC in patients with connective tissue disease [6, 7]. Thus, while it is a useful screening method, it is important to note that the margin of error in accuracy and the range of sensitivities present in echocardiographic estimation of RVSP in the relatively small population of individuals affected by SLE-associated PAH can considerably impact the ability to determine the true prevalence of this condition.

Molecular mechanisms contributing to the pathophysiology of PAH involve fibroblast and endothelial cell dysfunction that results in impaired production of vasodilators (including nitric oxide (NO) and prostacyclin) and overexpression of vasoconstrictors such as endothelin. These molecular derangements affect vascular tone and promote pathological vascular remodeling leading to pulmonary arterial vasoconstriction, in situ thrombosis, and occasionally complex plexiform lesions [8]. As the disease progresses, vascular remodeling and fibrosis eventually cause RV dilation and failure [1, 9]. In PAH patients with SLE, macrophages, lymphocytes, antinuclear antibodies, and complement have been identified histologically in the pulmonary vasculature [10].

In some SLE patients, there is overactivation of transcription factors known to be pathophysiologically relevant in idiopathic PAH such as hypoxia inducible factor-1 alpha (HIF-1a). There are also increased anti-endothelial cell antibodies, which lead to increased release of endothelin [4, 11]. Other relevant autoantibodies are anti-cardiolipin and anti-RNP antibodies, which have a positive correlation with the diagnosis of PH by echocardiography [11]. Despite these associations, it is unclear whether the presence of these autoantibodies has a direct mechanistic influence on the pathogenesis of PAH.

At the tissue level, vasculitis and thrombosis seen in SLE may contribute directly to vascular remodeling and damage. Furthermore, pulmonary venous hypertension from left ventricular dysfunction, hypoxic vasoconstriction from chronic hypoxemic lung disease, thromboses related to antiphospholipid antibody syndrome, and venoocclusive processes related to the hypercoagulable state in SLE may contribute indirectly to the development of PH [4].

Treatment options for PAH target three main molecular vascular derangements pathways [12]. The NO pathway is targeted by PDE-5 inhibitors and guanylate cyclase stimulators (sGC). The endothelin-1 pathway is targeted by endothelin-receptor antagonists (ERAs), and the prostacyclin pathway is targeted by synthetic prostacyclins and prostacyclin analogues, called “prostanoids.” PDE-5 inhibitors work by preventing PDE-5 from impairing the cyclic-GMP mediated vasodilatory function of NO. Guanylate cyclase stimulators increase cyclic-GMP (the downstream molecule of NO) and may function even in the absence of NO. ERAs inhibit endothelin, which is a potent vasoconstrictor overexpressed in PAH. Prostanoids work via a cyclic-AMP mediated relaxation of vascular smooth muscle causing vasodilation [12]. These four classes of drugs, though currently approved for treatment of PAH, are not approved for therapy in WHO groups 2–5 PH where these medications may cause harm in some cases (except for sGC which is also approved in WHO group 4 PH).

PAH is associated with other connective tissue diseases, such as systemic sclerosis and mixed connective tissue disease, and therapy aimed specifically at PAH may be sufficient in those cases. In contrast, patients with SLE and PAH often have worsening of PAH during SLE flares. In these patients presenting with active SLE and evidence of RV failure, induction immunosuppression followed by maintenance regimens significantly improved hemodynamic parameters [10]. In these studies, immunosuppressive therapy with monthly IV pulses of 600 mg/m^2^ of cyclophosphamide for six months plus prednisone 0.5–1 mg/kg/day for four weeks with a slow taper led to significant reduction in mPAP, improvement in cardiac index, and reduction in PVR [10].

In subgroup analyses, responders to this immunosuppressive approach were more likely to be anti-dsDNA and anti-Smith antibody positive and also had higher disease activity at the time of treatment. For those SLE patients with worse functional classification, a combination of the immunosuppressive strategy with cyclophosphamide and prednisone plus PAH-specific therapy with a prostanoid, ERA, or PDE-5 inhibitor resulted in improved hemodynamic outcomes compared to immunosuppressive therapy alone [10]. The importance of the benefit of combined SLE- and PAH-directed therapy is underscored by the finding that 3-year survival from time of diagnosis in patients with SLE-related PAH is 74% and is significantly better than the 3-year survival for patients with the more prevalent systemic sclerosis-associated PAH (47%) [13].

According to the Registry to Evaluate Early and Long-Term Pulmonary Arterial Hypertension Disease Management (REVEAL) risk score, this patient had a one-year mortality rate of 15–30% based on having WHO functional class IV, PAH associated with connective tissue disease, and a pericardial effusion [14]. In accordance with recent guidelines, a parenteral prostanoid can be used for treatment in this case [15]. She agreed to start a continuous infusion of treprostinil with informed consent after a patient-centered discussion of the importance of adherence as well as the risks, benefits, and alternatives (including ERAs which would have required her to get monthly documented pregnancy testing).

She was also given 1 gram of methylprednisolone daily for three days, and IV cyclophosphamide 500 mg/m^2^ to treat the acute SLE exacerbation, which had likely precipitated the worsening of her PAH. Hydroxychloroquine was restarted at 200 mg BID as was a gradual steroid taper. A similar regimen of immunosuppression in 2012, when she presented with SLE flare manifested by cutaneous vasculitis and PAH, resulted in complete recovery from acute right heart failure, without the need for pulmonary vasodilator therapy. At that time, she presented earlier in the course of a PAH exacerbation and thus had a better WHO functional class. Previous studies demonstrate that an immunosuppressive regimen alone without specific pulmonary vasodilators may be sufficient for SLE patients with less severe (better functional class) PAH exacerbations [10].

The combination of immunosuppressive therapy and PAH-specific therapy resulted in rapid improvements in her hemodynamic and respiratory status. The dosage of treprostinil was titrated to efficacy; she gradually was weaned off supplemental oxygen and inotropic support. She was discharged home after a 5-week hospitalization. Discharge medications included prednisone 1.5 mg/kg/day, hydroxychloroquine 200 mg BID, sildenafil 80 mg TID, and treprostinil by subcutaneous infusion at 49 ng/kg/min.

When seen in follow-up, her renal and hepatic failure had completely resolved. Her treprostinil infusion rate was increased to achieve further improvement in RV function. Laboratory studies also showed normalization of complements and a decrease in the anti-dsDNA level (Table 2). She was transitioned to prednisone 40 mg daily and continued on hydroxychloroquine 200 mg BID.

Despite the initial recovery from a life-threatening SLE-related PAH exacerbation, and multiple efforts to educate the patient about her life-threatening disease and the high mortality associated with abrupt discontinuation of prostanoid infusion, her subsequent clinical course was complicated by missed follow-up appointments and by nonadherence to her therapies, including infused prostanoid therapy. Five months after her presentation, she was brought to the ER in cardiopulmonary arrest and was unable to be resuscitated.

An important take-away message of this case report is the prompt recognition of severe PAH as a cause for progressive exertional dyspnea and hemodynamic instability in patients with rheumatologic diseases such as SLE. TTE provides a noninvasive bedside screening technique. Also important is the variable course that SLE-associated PAH can take in the same patient and the implications for instituting the most effective treatment approach with each episode.

There are effective, evidence-based therapeutic options for treating both PAH itself with vasodilator therapy (e.g., a prostanoid, ERA, or PDE-5 inhibitor) and the SLE flares leading to PAH exacerbation with immunomodulators (e.g., high-dose corticosteroids and cyclophosphamide). The cause of PAH in SLE is often a result of concomitant molecular and tissue-level factors (autoimmune disease activity, endothelial damage, and thrombosis). Given the difficulty in ascribing the etiology of an exacerbation to any one factor, treatment strategies employing both immunomodulators and pulmonary vasodilators to target the multiple convergent pathophysiologic pathways are likely more beneficial than therapy with a single pharmacologic modality. More trials are needed to delineate the relative role of immunosuppressive therapy in SLE-associated PAH compared with pulmonary vasodilator therapy, especially as recent survival data shows that pulmonary vasodilator therapy is pivotal in SLE-associated PAH.

A multidisciplinary approach involving pulmonologists and/or cardiologists with experience in PH and rheumatologists with an understanding of the complex management of PAH in SLE and other connective tissue diseases is important to therapeutic success. Equally important is a collaborative partnership with the patient in carrying out the treatment plan. As demonstrated by the literature reviewed herein, early recognition of the symptoms of PAH in SLE allows for prompt and effective intervention that may favorably impact the patient's functional status and survival.

## Figures and Tables

**Table 1:** World Health Organization (WHO) classification schemes for pulmonary hypertension (PH) and functional class (FC). The examples given for each WHO PH group are not comprehensive but offer representations of disease processes in each category. The patient in this report is group 1 PH with FC IV.

Category	Characteristics
All groups of PH	mPAP of ≥25 mmHg at rest, PVR of >240 Dynes-sec/cm^5^, PAWP ≤15 mmHg (except for group 2 PH where PAWP ≥15 mmHg).
Group 1 PH	Pulmonary *arterial* hypertension (PAH). It includes idiopathic PAH, PAH from genetic mutations, medications, HIV, portal hypertension, congenital heart disease, and schistosomiasis. It also includes PAH associated with connective tissue diseases such as SLE and systemic sclerosis.
Group 2 PH	Pulmonary *venous* hypertension (left-sided heart disease/failure).
Group 3 PH	PH owing to chronic lung diseases and/or hypoxemia (e.g., chronic obstructive pulmonary disease, sleep disordered breathing, and interstitial lung diseases).
Group 4 PH	PH from chronic thromboembolic disease.
Group 5 PH	PH occurring in several miscellaneous conditions whose association with PH is poorly understood (e.g., sarcoidosis, lymphangioleiomyomatosis, and Langerhans cell histiocytosis).

FC I	No symptoms with ordinary physical activity.
FC II	Fatigue, dyspnea, chest pain, or syncope with ordinary physical activity.
FC III	Symptoms that develop with less than ordinary physical activity.
FC IV	Symptoms with any physical activity, or while at rest.

**Table 2:** Laboratory assessment of immunologic and inflammatory disease activity at admission and at follow-up 8 weeks later showing serologic phenotype and response to therapy.

Immunologic parameter (units, where applicable)	Reference range	Admission value	Postdischarge follow-up value
ESR (mm/hr)	0–20	35	46
CRP (mg/L)	0.1–3.0	27.9	9.2
C3 (mg/dL)	88–145	33	128
C4 (mg/dL)	16–39	<10	22
ANA titer	<1 : 40	>1 : 10,240^∧^	1 : 2,560
Anti-dsDNA Ab^*∗*^ (IU/mL)	<12.5	25.7	<12.5
Anti-centromere Ab	<1 : 40	<1 : 40	
Anti-SCL70	Negative	Negative	
Anti-La Ab	Negative	Negative	
Anti-Ro Ab	Negative	Positive	
Anti-Smith Ab^*∗*^	Negative	Positive	
Anti-RNP Ab^*∗∗*^	Negative	Positive	
Anti-cardiolipin Ab^*∗∗*^ (CU)	<20	10.6	
Beta-2 glycoprotein (CU)	<20	14.9	

^∧^Speckled pattern. ^*∗*^Patients positive for anti-dsDNA and anti-Smith had better response to immunosuppressive therapy during an SLE-associated PAH flare [10]. ^*∗∗*^Anti-RNP and anti-cardiolipin positivity correlates with evidence of PH on echocardiogram [11]. Ab: antibody; ESR: erythrocyte sedimentation rate; CRP: C-reactive protein; ANA: antinuclear antibody; dsDNA: double-stranded DNA; RNP: ribonucleoprotein.

**Table 3:** Hemodynamic parameters from right heart catheterization of the patient after successful treatment of 2012 episode of PAH compared to the current presentation of severe PAH and cardiogenic shock.

Hemodynamic parameter (units)	Reference range	Symptom-free baseline (2012, posttreatment)	Current PAH exacerbation^*∗*^
RAP (mmHg)	1–6	1	7
PAWP (mmHg)	6–15	4	4
PAP (mmHg)	20–30/10–15	27/11	86/51
Mean PAP (mmHg)	10–20	17	62
CO (L/min)	4–8	7.8	4.2
CI (L/min/m^2^)	2.6–4.2	4.9	2.6
PVR (Dynes-sec/cm^5^)	≤240	128	784

RAP: right atrial pressure; PAWP: pulmonary artery wedge pressure; PAP: pulmonary artery pressure designated here as systolic/diastolic; CO: cardiac output; CI: cardiac index; PVR: pulmonary vascular resistance. ^*∗*^Note: measurements made while patient was on vasopressors and inotropes for clinical and echocardiographic evidence of acute cardiogenic shock given the need for emergent hemodynamic support and stabilization before the RHC could be performed safely.
